# Undergraduate training in Psychiatry: World perspective

**DOI:** 10.4103/0019-5545.37316

**Published:** 2007

**Authors:** R. Srinivasa Murthy, S. Khandelwal

**Affiliations:** Professor of Psychiatry (Retd.), NIMHANS, Bangalore, India; * Department of Psychiatry, All India Institute of Medical Sciences, New Delhi, India

## HISTORICAL DEVELOPMENT OF MENTAL HEALTH SERVICES

During the last three centuries, there has been major shifts in the way mentally ill are viewed and cared for all over the world. The changes during the last 50 years are most significant. From a situation of considering the “mad” as “bad” and incarceration in jails and asylums, there is now recognition of the human rights of the mentally ill. From jails and asylums, the care of the mentally ill persons has moved to the community. Another important development is the care providers. Current approach to care in the community includes, besides psychiatrists, other mental-health professionals, primary-care doctors, family members, volunteers and the ill persons. The driving forces towards these changes have been many: the recognition of the wide range of mental disorders, the high prevalence of mental disorders in the community, the availability of a variety of interventions (pharmacological and nonpharmacological), the demonstration of the effectiveness.

## HUMAN RESOURCES FOR HEALTH CARE

There is an international focus on human resources for health care. The theme of the World Health Report 2006 (WHR 2006) was Working Together for Health. The WHR 2006 begins with the following observation:

*“In this decade of the 21^st^ century, immense advances in human well-being coexist with extreme deprivation. In global health we are witnessing the benefits of new medicines and technologies. But there are unprecedented reversals. Life expectancies have collapsed in some of the poorest countries to half the level of the richest - attributable to the ravages of HIV/AIDS in parts of sub-Saharan Africa and to more than a dozen “failed states”. These setbacks have been accompanied by growing fears, in rich and poor countries alike, of new infectious threats such as SARS and avian influenza and “hidden” behavioural conditions such as mental disorders (emphasis added) and domestic violence*.”(xv) (emphasis added).

### Further, the report states

The ultimate goal of health workforce strategies is a delivery system that can guarantee universal access to health care and social protection to all citizens in every country. *There is no global blueprint that describes how to get there - each nation must devise its own plan. Effective workforce strategies must be matched to a country's unique situation and based on social consensus* (emphasis added). (p.119)

The human resources for mental health care are grossly inadequate in the developing countries, as presented by the WHO Atlas document in 2005. In the Indian context, the development of appropriate human resources for health in general is receiving serious attention. For example, the setting up of the Public Health Foundation of India in 2006 is a good example of this concern.

Both authors come from a background of community mental health and experience of training and working with primary health-care doctors. The effort is to present the “world” perspective (from the World Health Report, the World Psychiatric Association (WPA) guidelines, experiences of different countries) and identify some issues relevant to undergraduate education in India.

### World Health Report 2001

The World Health Report 2001 makes 10 overall recommendations. The first of this is “Provide treatment in primary care.” The management and treatment of mental disorders in primary care is a fundamental step which enables the largest number of people to get easier and faster access to services. It is to be recognized that many are already seeking help at this level. This not only gives better care but also cuts wastage resulting from unnecessary investigations and inappropriate and nonspecific treatments. For this to happen, however, general health personnel need to be trained in the essential skills of mental health care. Such training ensures the best use of the available knowledge for the largest number of people and makes possible the immediate application of interventions. Mental health should therefore be included in training curricula, with refresher course to improve the effectiveness of the management of mental disorders in general health services.

Integration of mental health care into general health services, particularly at the primary health-care level, has many advantages. These include less stigmatization of patients and staff, as mental and behavioral disorders are being seen and managed alongside physical health problems; improved screening and treatment, in particular, and improved detection rates for patients presenting with vague somatic complaints which are related to mental and behavioral disorders; the potential for improved treatment of the physical problems of those suffering from mental illness and vice versa; and better treatment of mental aspects associated with “physical” problems. For the administrator, advantages include a shared infrastructure leading to cost-efficiency savings, the potential to provide universal coverage of mental health care and the use of community resources which can partly offset the limited availability of mental health personnel.

The specific ways in which mental health should be integrated into general health care will, to a great extent, depend on the current function and status of primary-, secondary- and tertiary-care levels within countries' health systems.

For integration to be successful, policy makers need to consider the following: general health staff must have the knowledge, skills and motivation to treat and manage patients suffering from mental disorders; there need to be sufficient numbers of staff with the knowledge and authority to prescribe psychotropic drugs at primary and secondary levels; basic psychotropic drugs must be available at primary and secondary levels; mental health specialists are required to provide support to, and monitor, general health-care personnel; effective referral links between primary, secondary and tertiary levels of care need to be in place; funds must be redistributed from tertiary to secondary and primary levels of care or new funds must be made available; and recoding systems need to be set up to allow for continuous monitoring, evaluation and updating of integrated activities.

## WORLD PSYCHIATRIC ASSOCIATION RECOMMENDATIONS

In 1998, the WPA, along with the World Federation of Medical Education (WFME) through a core curriculum committee, developed detailed guidelines for the “Core Curriculum in Psychiatry for Medical Students.” One of the authors (RSM) was a member of the core curriculum committee. The recommendations present the world view of the subject. The main recommendations are given below. (The full report is available from the WPA website.)

That Psychiatry should occupy a major part in the medical curriculum is now generally agreed. There are three reasons for this agreement. First, the general approach of Psychiatry which stresses the unity of body and mind is important in the whole of medical practice. Secondly, skills that are learned in Psychiatry are important for all doctors: for example, the ability to form a good relationship with a patient, to assess the mental state and to impart distressing information. Thirdly, psychiatric problems are common among patients seen by doctors working in all branches of Medicine: for example, it is known that among outpatients attending specialist clinics, about 15% of those given a diagnosis have an associated psychiatric disorder; and an average of 20-30% of those given no medical diagnosis have a psychiatric disorder. Psychiatric disorders are even more frequent among patients attending general practice. Therefore, all future doctors must know about these psychiatric problems, not only because they are common but also because their management involves much medical time and resources and gives rise to many serious incidents.

## THE PROPOSED CURRICULUM

The core component in Psychiatry in the curriculum described in the WPA/WFME Report is the minimum that is required by medical students who, after qualification, will enter further training whether they are to work as specialists or in primary care. In many countries, doctors who have chosen a career in primary care (general practice) receive a further period of training after graduation; and in most of these countries, this training extends their psychiatric skills. In countries with no formal training for primary care doctors (general practitioners), the teaching in Psychiatry described in this report needs to be supplemented by a module containing the additional material that is essential for management of the psychiatric morbidity encountered in general practice. This module will need to be developed locally to take account of the special circumstances of practice in the country. The report describes the minimum requirements in Psychiatry for medical students who will enter further training, whether they are to work as specialists or in primary care.

## ATTITUDE OBJECTIVES

Since most students will not enter Psychiatry, the acquisition of appropriate attitudes is of primary importance. It is important that the objective of imparting these attitudes is in the teacher's mind throughout his/her interaction with students. Most of the attitudes to be acquired while learning Psychiatry do not differ from those needed to practice the rest of Medicine. The extent to which these attitudes are emphasized to students in the Psychiatry program, rather than during the periods for other subjects, will vary from one medical school to another. However, each school should have a clear plan that ensures that the necessary attitudes have been acquired by the time the students graduate. It is important that students develop appropriate attitudes towards Psychiatry as a medical discipline. These attitudes will be encouraged, particularly during the teaching of Psychiatry, but it is important that they are not negated during the teaching of other subjects. It is important that attitudes are not merely expressed verbally by students but are also internalized, directing how students respond to patients and their colleagues. Each of the attitudes listed below should be translated into corresponding action.

### Attitudes concerned with medical practice generally

Students should recognize that the profession of Medicine requires lifelong learning and show capacity for critical thinking and constructive self-criticism; be able to tolerate uncertainty and be open-minded to the views of others and be able to work constructively with other health professionals.

### Attitudes towards patients and their families

Students should respect patients and understand their feelings; recognize the necessity of good doctor-patient relationships; appreciate the value of the developmental approach to clinical problems, emphasizing the stages of the life cycle and longitudinal perspective of illness; recognize the importance of the family and the wider environment of the patient and attitudes towards Psychiatry as a medical discipline

Students should recognize the value of Psychiatry as a medical discipline; integrate humanistic, scientific and technological aspects of knowledge of Psychiatry and recognize the importance of the promotion of mental health and the prevention of psychiatric disorders.

## KNOWLEDGE OBJECTIVES

The knowledge objectives of Psychiatry include psychiatric symptoms and syndromes; psychological aspects of medical disorders (“psychological medicine”); and psychosocial issues, including stigma. Psychiatric symptoms and syndromes and their treatment are to be taught and learned in the context of an integrated biological, psychological and social approach. Knowledge objectives can be formulated in broad terms or as a detailed curriculum. A detailed list may be important not only to guide teachers and students but also to indicate to the deans and curriculum committees of the medical school, the substantial factual basis of Psychiatry and the resources needed to teach this.

Whatever level of detail is chosen concerning each individual disorder, collectively these should provide opportunities to (a) illustrate the approach to etiology in psychiatry; (ii) discuss attitude objectives and “teaching of skills” objectives; (iii) provide instruction concerning action that should be taken.

## SKILLS OBJECTIVES

The skills required by medical students range from those with which they need only be familiar (in the sense of being aware that they are practiced by others, e.g., dynamic psychotherapy) to those skills which students are expected to utilize competently themselves. Many of the skills students learn in Psychiatry overlap with those learnt in the other branches of Medicine. The stage in the curriculum at which the various skills should be learnt is a matter for the curriculum committee of the medical school to decide.

### Skills to be acquired include the following:

Doctor-patient interpersonal skills include the skills of “active listening”; empathy; nonverbal communication; opening, controlling and closing an interview.

Information gathering skills include taking of the history of patient's complaints and a life history; carrying out a physical examination, taught also in other parts of the curriculum; these also includes skills necessary to assess the functioning of the patient's family and the family's ability to contribute to the patient's care. Information-evaluation skills include selecting the crucial pieces of information for making a diagnostic formulation and undertaking a differential diagnosis, making a personality assessment, evaluating the role of personal and social factors in the patient's behavior, formulating a plan of management which includes the points at which referral to a specialist will be appropriate. Information-giving skills include passing information to patients to promote health, explaining the implications of a diagnosis and informing patients about the beneficial and potential adverse effects of treatment. Reporting skills include reporting verbally or in writing to medical colleagues; lay people, including the relatives of patients, nonmedical agencies involved in the care of patients and promoting public education. Treatment skills include promotion of compliance with prescribed treatment, basic prescribing skills for the psychiatric disorders commonly encountered by nonpsychiatrists, recognizing adverse effects of treatment and distinguishing them from symptoms of illness.

## GUIDELINES FOR TEACHING AND LEARNING OF PSYCHIATRY

Learning should self-directed, problem-based learning, with locally produced teaching aids along with exposure to a range of patients in different settings; and integrated psychiatric teaching and learning in the curriculum.

### Assessment

Teaching methods should be evaluated by students, thus helping individual teachers to improve their performance and to upgrade the teaching programs as a whole. A distinction is to be made between two types of assessments. Formative assessment is designed to give feedback to the student about his progress as he proceeds. Summative assessment is carried out at the end of the courses for purposes of grading. Both teachers and students should evaluate each course; the latter assessments are particularly valuable.

### The training of teachers

It is important that teaching is recognized as an important activity within the medical school. Also, the financial rewards for teaching should be commensurate with those for clinical work if teachers are to be encouraged and retained. Teaching staff are required to have an interest in teaching and have to realize that they require to be trained for their role as teachers. It is thus important that account is taken of interest in teaching when teaching staff are appointed. University departments should give high priority to teacher training, within the medical school, so that staff have educational expertise as well as clinical and research competence. There should be an educational development program to extend all teachers' understanding of the teaching-learning process, and it should be updated regularly. Educational resources should be provided: educational resource persons, educational literature and regular seminars and workshops. The teachers of Psychiatry should participate in the educational committee in the medical school responsible for the curriculum as a whole.

## ADDITIONAL TEACHING FOR PRIMARY HEALTH CARE

In developed countries medical students graduate as generic doctors, who can enter general practice only after further training. In other countries students can work in general practice as soon as they qualify fully without this additional training in general practice. In the latter countries medical students need to receive additional teaching in Psychiatry during the medical study period, since psychiatric disorders form such a large part of the work of primary-care doctors. This additional psychiatric training in developing countries should extend across the medical curriculum as a whole. It should also continue, after graduation, as part of in-service training and continuing medical education (CME).

The teachers in the countries concerned will be able to decide the necessary content of this additional preparation for general practice responsibility directly after qualification. In addition to the knowledge content, the skills needed to diagnose psychiatric problems within a system appropriate for primary care will be important, as also the skills needed for treatments used most often by general practitioners and the knowledge of when to refer to specialist services. Attitudes that will promote mental health and reduce stigma have also to be acquired. A substantial part of this additional teaching should take place in the community settings in which students are likely to work when qualified, and teamwork skills necessary for the doctor to do his work in conjunction with nonmedical staff are critically important. The international classification of diseases (ICD) classification for primary care is a useful guide to the additional teaching needed for primary care.

### Time and resources

It is an important responsibility of psychiatric teachers to convince the medical faculty of the value of Psychiatry in the general medical curriculum. The case is made by (a) the frequency of psychiatric problems in the general practice of medicine, (b) the substantial factual basis of the subject and (c) the need to teach communication skills. When this importance has been accepted, the time needed to teach the subjects will follow. Exact figures about the amount of teaching hours that are required for the core curriculum depend in part on the amount of conjoined teaching with other departments and the extent of teaching of communication skills during other parts of the medical curriculum. The amount of time spent in the Psychiatry department will also depend on the other opportunities for teaching behavioral science and psychosocial aspects of Medicine in the curriculum as a whole. Corresponding resources need to be provided for this teaching.

In addition to this full-time study, two other periods of teaching are essential. First, opportunities to teach Psychiatry are required during other clinical attachments, especially in medicine, surgery and general practice attachments. Secondly, an adequate proportion of time allocated for lectures and seminars in the curriculum should be allocated to Psychiatry and mental-health issues. Such teaching should be scheduled at several times in the curriculum, selected by the Teaching and Learning Committee of the medical school, according to the opportunities available and the skills of the teachers. With these provisos in mind, a period of eight weeks is required to teach Psychiatry; however, some of this teaching can be outside the single block of a psychiatry attachment, though the latter should never be less than four weeks. Corresponding resources need to be allocated to provide adequate teaching and learning during this period. The timing of the full-time attachment within the course is generally best in the second clinical year, provided that some additional time for teaching is allocated in the first and third years.

## INTERNATIONAL SITUATION

In the USA, Behavioral Sciences are taught in the first year of undergraduate studies. During the first two years, there are about 60 hours of teaching in various psychosocial areas. In the third year, 30 hours are devoted to practical teaching of Psychiatry. In the fourth year, there is a full-time posting of 8 weeks of Psychiatry clerkship compared to 8 weeks each allotted for Obstetrics and Pediatrics and 12 weeks for both Medicine and Surgery.

In Denmark, the teaching of mental disorders started in 1902 and was well established in 1912. During the fifties it acquired the status of a major clinical subject, rising to third place after Surgery and Medicine and ahead of Pediatrics and Obstetrics and Gynecology. Now there are approximately 240 hours of Psychiatry teaching in a six-year course, comprising about 7% of the total time. It is a major clinical discipline with a qualifying examination at the end of the course.

In Britain, Psychiatry established its place in medical education in the forties. However, it went through major changes in the seventies following the General Medical Council's recommendations in 1967 regarding medical education, which re-emphasized the importance of Behavioral Sciences and Psychiatry in medical teaching and practice. Currently, 80 hours are devoted to the behavioral science course during basic medical science teaching. During the clinical course, students first learn interview skills and psychiatry history-taking once a day during the 36 weeks and then attend a full-time Psychiatry clerkship for 3 months. This is usually followed by a university examination as in other subjects.

There is a greater emphasis in Malaysia on the teaching of Psychiatry since the introduction of a new curriculum in the late seventies. Psychiatric aspects of various clinical disorders are discussed from the first year, followed by separate courses in Community Psychiatry and General Psychiatry. Most of the clinical teaching takes place in the fourth and fifth years of the course and consists of lectures, seminars and tutorials. Students are provided the opportunity to develop skills in history-taking and interview techniques. They are required to submit in detail five case histories, as well as a discussion on management. At the end of posting, they are formally assessed. The total teaching involves approximately 100 hours (Jayaswal, 2007, personal communication).

In the last three decades, many newly independent African countries have started medical colleges. Although their curricula provide for a substantial time for the teaching of Psychiatry, owing to inadequate resources and shortage of trained manpower the courses are not always held regularly. Most psychiatrists are expatriates whose mobility is very high. In Ethiopia, where the second author has had firsthand experience, there is a provision for 20 hours of teaching of Behavioral Sciences during the first two years of the medical course, followed by a full-time, eight-week posting in Psychiatry during the fourth and final years. Unfortunately owing to shortage of staff, this has been reduced to only six weeks. However, it remains one of the major seven subjects taught and examined in the final year, the others being Medicine, Surgery, Gynecology and Obstetrics, Pediatrics, Ophthalmology and Community Medicine. The broad aims of the course are that students should learn the importance of psychological factors in social and clinical context, diagnose psychiatric illness and be able to give at least primary care to serious conditions such as psychosis, depression, epilepsy and mental retardation. The specific objectives are to enable students (i) to recognize major psychiatric disorders in the community and be able to treat some of them and refer others to the appropriate agencies, (ii) to deal with psychiatric emergencies and (iii) to understand the role of psychological factors in physical disorders.

Closer home, Sri Lanka and Nepal have expanded the duration of Psychiatry teaching, revised undergraduate Psychiatry curriculum and made Psychiatry a qualifying subject for M.B.B.S. examination.

## CHALLENGES IN THE INDIAN SITUATION

Ever since the time of Bhore Committee in 1946, many committees and task forces have uniformly emphasized the importance of reforms in the undergraduate Psychiatry teaching by (i) increasing the amount of training in Psychiatry, (ii) making Psychiatry an independent subject for teaching and examination and (iii) revising the Psychiatry curriculum at the undergraduate level.

It is not a secret that even in the 21^st^ century, as many as 25-30% of medical colleges in India do not have independent departments of Psychiatry. The exposure of medical students to mental disorders is limited to some visits to the mental hospitals, but inhumane conditions there result in creating fears among medical students rather than developing their interest in mental-health problems. Till date, the M.B.B.S. syllabus prescribed by the Medical Council of India (www.mciindia.org/know/rules/rules-mbbs.htm as accessed on 17.6.07) devotes only 20 lectures to Psychiatry and a two-week posting of 3 hours/day in Psychiatry. Psychiatry is taught as an allied discipline of Medicine, though it has an ambitious curriculum (see Appendix). This is in spite of recommendations of various committees, including one by the Medical Council of India (MCI) itself, to make Psychiatry a separate subject with increased allocation of time.

In India, the following initiatives are required on an urgent basis.

1. Revising the curriculum and enhancing the quality of training
  In parallel with increasing the time allocated to Psychiatry teaching, there has been felt a greater need of introducing social and behavioral sciences in the training of medical undergraduates to make them more socially aware and responsive and develop a holistic community-oriented approach.
  General epidemiological surveys in primary-care setting have indicated that as many as 30% of patients attend health services primarily for a mental-health problem. General practitioners are already sharing the burden of mental-health problems. However, to increase their effectiveness in identifying and managing common mental and behavioral health problems in the community, including screening and early intervention for tobacco, alcohol and other drug use, they must be provided quality training at the undergraduate level itself with frequent refresher courses as part of continuing medical education subsequently.
  The training curriculum should also include application of evidence-based psychosocial strategies, skill-building in the areas of administration and management, policy development and research methods.
2. Strengthening teachers of Psychiatry
  Technological revolution has been sweeping the world, bringing rapid changes in our knowledge and beliefs. Medical sciences too have witnessed such a revolution, demanding rapid changes in the content as well as method of its delivery. This may put a greater stress on the teachers who, in a typical Indian medical college, may already be burdened with high clinical workload and administrative responsibilities. Teachers must be involved regularly in workshops, etc., to enhance their teaching skills by application of newer technology like audiovisual media, computers, internet and other tools of information technology.
3. Research in mental-health care
  Mental-health systems are undergoing major reforms in many countries. Spurred by de-institutionalization, the development of community-based services/primary-care services has been integrated into the overall health system. There is an ideological belief that alternative forms of community mental-health care that are not dependent on the specialist would be more cost-effective. There is now evidence, derived from controlled studies, demonstrating the effectiveness of such ideology. However, most of the research to date has been generated in the developed countries (WHR, 2001). Research is needed in the developing countries to generalize this evidence and then guide reforms in mental-health care. It goes without saying that for this to happen at sustained and practical level, we need to strengthen the undergraduate teaching of Psychiatry, enabling a community-based physician to deliver the essential mental-health care.

### Future directions

It is abundantly clear that psychiatrists alone cannot meet the mental-health needs of this country. Moreover, lately, there has been a huge demand for psychiatrists in countries like Britain, Australia and New Zealand, leading to regular migration of trained psychiatrists from India to these countries. Thus there will remain a huge gap between demand and supply of psychiatrists within the country. If India has to effectively deal with its mental-health burden, it has to develop alternate models of care as in other countries, including developed countries. Many international organizations like World Federation of Medical Education and World Psychiatric Association have developed a core curriculum in Psychiatry that can be used as a template by various countries for developing their own curriculum. Indian Psychiatric Society must take lead in this endeavor to bring about necessary changes in psychiatric teaching at the medical council of India (MCI) level. The benefits of strengthening the psychiatric component in undergraduate medical education extend far beyond the primary objective of equipping our future doctors with better psychiatric skills, considering the fact that nearly 15,000 trained doctors are added every year in India who could do wonders in managing mental-health needs of the country.

## APPENDIX: MCI GUIDELINES FOR PSYCHIATRY

**Goal**

The aim of teaching the undergraduate student in Psychiatry is to impart such knowledge and skills that may enable him to diagnose and treat common psychiatric disorders, handle psychiatric emergencies and to refer complications/unusual manifestations of common disorders and rare psychiatric disorders to the specialist.

**Objectives**

- Knowledge
  At the end of the course, the student should be able to:
  1. comprehend nature and development of different aspects of normal human behavior, like learning, memory, motivation, personality and intelligence;
  2. recognize differences between normal and abnormal behavior;
  3. classify psychiatric disorders;
  4. recognize clinical manifestations of the following common syndromes and plan their appropriate management: organic psychosis, functional psychosis, schizophrenia, affective disorders, neurotic disorders, personality disorders, psychophysiological disorders, drug and alcohol dependence, psychiatric disorders of childhood and adolescence;
  5. describe rational use of different modes of therapy in psychiatric disorders.
- Skills
  The student should be able to:
  1. interview the patient and understand different methods of communications in patient-doctor relationship;
  2. elicit detailed psychiatric case history and conduct clinical examination for assessment of mental status;
  3. define, elicit and interpret psychopathological symptoms and signs;
  4. diagnose and manage common psychiatric disorders;
  5. identify and manage psychological reactions and psychiatric disorders in medical and surgical patients in clinical practice and in community setting.
- Integration
  Training in Psychiatry should prepare the students to deliver preventive, primitive, creative and rehabilitative services for the care of patients both in the family and community and to refer advance cases to a specialized psychiatry/mental hospital. Training should be integrated with the departments of Medicine, Neuroanatomy, Behavioral Sciences and Forensic Medicine.
